# LOTUS Inhibits Neuronal Apoptosis and Promotes Tract Regeneration in Contusive Spinal Cord Injury Model Mice

**DOI:** 10.1523/ENEURO.0303-18.2018

**Published:** 2018-12-14

**Authors:** Shuhei Ito, Narihito Nagoshi, Osahiko Tsuji, Shinsuke Shibata, Munehisa Shinozaki, Soya Kawabata, Kota Kojima, Kaori Yasutake, Tomoko Hirokawa, Morio Matsumoto, Kohtaro Takei, Masaya Nakamura, Hideyuki Okano

**Affiliations:** 1 Department of Physiology, Keio University School of Medicine, Tokyo 160-8582, Japan; 2 Department of Orthopaedic Surgery, Keio University School of Medicine, Tokyo 160-8582, Japan; 3 Molecular Medical Bioscience Laboratory, Yokohama City University Graduate School of Medical Life Science, Yokohama 230-0045, Japan

**Keywords:** axonal regeneration, LOTUS, neuronal protection, Nogo receptor, spinal cord injury, transgenic mice

## Abstract

Nogo receptor-1 (NgR1) signaling is involved in the limitation of axonal regeneration following spinal cord injury (SCI) through collapsing the growth cone and inhibiting neurite outgrowth. Lateral olfactory tract usher substance (LOTUS), a NgR antagonist, suppresses these pathological conditions. A previous report demonstrated the positive effects of LOTUS expression on motor function through raphespinal tract regeneration using pan-neuronally LOTUS-overexpressing transgenic mice. However, this report used a hemi-section model, which does not represent the majority of clinical SCI cases, and lacked a detailed histological analysis of other descending tracts. To determine the true therapeutic effects of LOTUS, we used a more clinically relevant contusive SCI model in female transgenic mice. Definitive tracing analyses revealed that LOTUS promoted the extensive regeneration of the reticulospinal tract across the lesion site and suppressed axonal dieback of corticospinal tract (CST). A significant increase in raphespinal tract fibers was seen from the subacute to the chronic phase after the injury, strongly suggesting that LOTUS promoted translesional elongation of this tract. Furthermore, histological analyses revealed that LOTUS had a neuroprotective effect on the injured spinal cord through suppressing cellular apoptosis during the acute phase. These neuroprotective and regenerative effects contributed to significant motor functional recovery and restoration of the motor evoked potential (MEP). Therefore, LOTUS application could prove beneficial in the treatment of SCI by promoting axonal regeneration of some descending fibers, reducing axonal dieback of CST fibers and encouraging motor function recovery.

## Significance Statement

Axonal regeneration following spinal cord injury (SCI) is extremely limited due to Nogo receptor (NgR) signaling, which inhibits neurite outgrowth and collapses growth cones. Lateral olfactory tract usher substance (LOTUS), a NgR antagonist, suppresses this inhibition by NgR. Here, we evaluated the therapeutic effects of LOTUS using a clinically relevant contusive SCI model. Our data revealed that LOTUS promoted extensive translesional regeneration of the reticulospinal and raphespinal tracts and rostral regeneration of the corticospinal tract. Furthermore, LOTUS suppressed cellular apoptosis during the acute phase, and these neuroprotective and regenerative effects contributed to significant recovery of motor function and nerve conduction. Therefore, this study suggested that LOTUS could aid the treatment of SCI by promoting axonal regeneration and motor function recovery.

## Introduction

Natural recovery following spinal cord injury (SCI) is extremely limited in mammalian adults (Schwab, 2010) due to the inhibition of axonal regeneration controlled by Nogo receptor-1 (NgR1) signaling (Fournier et al., 2001). In the injured spinal cord, ligands of NgR1, such as Nogo (GrandPré et al., 2000), myelin-associated glycoprotein (MAG; McKerracher et al., 1994), oligodendrocyte myelin glycoprotein (OMgp; Wang et al., 2002b), B lymphocyte stimulator (BLyS; Zhang et al., 2009), and chondroitin sulfate proteoglycans (CSPGs; Silver and Miller, 2004), bind to NgR1, which interacts with coreceptor p75 (Wang et al., 2002a). Downstream, Ras homolog gene family, member A (Rho-A) and Rho-associated kinase (ROCK) are activated, causing growth cone collapse, thereby inhibiting neuronal regeneration (Domeniconi et al., 2002; Dickendesher et al., 2012).

Since NgR1 plays an important role in controlling neuronal outgrowth, the important role of its signal cascade in the treatment of SCI has been recognized. Previous reports have mainly focused on axonal regeneration of the corticospinal tract (CST; Lemon, 2008). However, the results from several studies using Nogo-deficient mice to evaluate its impact on CST fibers and motor function have been inconsistent (Kim et al., 2003; Simonen et al., 2003; Zheng et al., 2003). Mice lacking MAG or OMgp also failed to regenerate the CST following SCI (Bartsch et al., 1995; Ji et al., 2008). Kim et al. (2004) demonstrated locomotor recovery after SCI by inhibiting a common receptor of all ligands using NgR1-lacking mice. Interestingly, this animal model exhibited regeneration of the raphespinal tract, which plays an important role in the formation of connections with the motor neural circuit after SCI (Liang et al., 2015). Therefore, by blocking the downstream Rho-ROCK cascade, the NgR1 antagonist may enhance tract regeneration following SCI.

Recently, cartilage acidic protein-1B (Crtac1B) has been identified as a lateral olfactory tract usher substance (LOTUS), which is both a membrane and secreted protein that functions as an endogenous antagonist against NgR1 (Sato et al., 2011; Kurihara et al., 2012). LOTUS binds to NgR1 and inhibits all ligand proteins, resulting in decreased growth cone collapse and neurite outgrowth inhibition (Kurihara et al., 2014, 2017; Kawakami et al., 2018). In focal brain ischemia model mice, LOTUS overexpression on the contralateral side of the injury accelerated axonal sprouting that crossed the midline to the ipsilateral side, enhancing the neuronal plasticity of motor pathways (Takase et al., 2017). LOTUS overexpression was recently demonstrated to enhance the regeneration of raphespinal tract fibers across the lesion, thereby contributing to motor functional recovery (Hirokawa et al., 2017).

In the above paper, the authors used dorsal hemi-section to model SCI and observed tract fiber regeneration. However, transection injury is rarely observed in the clinical setting. In fact, a previous study advocated that contusive injury is more clinically relevant than complete or partial transection (Kwon et al., 2011). Therefore, the efficacy of LOTUS in preclinical studies should be evaluated using contusive injury models. Additionally, the previous study evaluated only raphespinal tracts and did not examine CST and reticulospinal tracts. Particularly, the reticulospinal tract is strongly involved in the neural networks that coordinate rhythmic stepping movements (Fink and Cafferty, 2016). Finally, although the aforementioned study illustrated an increase in 5-hydroxytryptamine (5-HT)-positive raphespinal tract axons in the chronic stage, whether this phenomenon was a result of axonal preservation or regeneration after SCI remains unclear. Antagonistic activity against NgR1 or Rho-A can suppress apoptosis following CNS injury (Dubreuil et al., 2003; Wang et al., 2008), implying that LOTUS, a NgR1 antagonist, could also exert its neuroprotective effect during the acute inflammatory phase following SCI.

In the present study, we evaluated the effect of LOTUS on spinal contusive injury using transgenic mice overexpressing LOTUS by specifically focusing on axonal regeneration in various tract fibers, such as the corticospinal, reticulospinal and raphespinal tracts, using tracing and various histologic methods. Moreover, we investigated the neuroprotective effect of LOTUS during the acute stage of SCI. This study is the first to demonstrate the beneficial effect of LOTUS in a clinically relevant traumatic SCI animal model.

## Materials and Methods

### Animals

LOTUS-overexpressing (LOTUS-Tg) mice were generated using the mouse synapsin-1 promoter, which selectively expresses neuron-specific LOTUS as previously described (Hirokawa et al., 2017). C57BL/6J mice were purchased from CLEA Japan, Inc. We used LOTUS-Tg and C57BL/6J mice (eight-week-old, female, 18–22 g) in this study. All experiments were approved by the ethics committee of Keio University and were in accordance with the Guide for the Care and Use of Laboratory Animals (National Institutes of Health, Bethesda, MD).

### SCI model

All mice were anesthetized by intraperitoneal injections of ketamine (60 mg/kg) and xylazine (10 mg/kg). The laminal arch of the vertebrae at the tenth thoracic level was removed, and the exposed dura mater was subjected to a 70-kdyn contusive SCI using a commercially available SCI device (IH Impactor, Precision Systems and Instrumentation) as previously described (Scheff et al., 2003). After SCI, 12.5 mg/kg ampicillin was administered intramuscularly.

### Western blot analysis

For intact mice, 4-mm-long samples of the spinal cord centered at the tenth thoracic level were used for analysis. Tissue samples were homogenized in lysis buffer, and 10-μg protein was electrophoretically separated on 4–20% gradient polyacrylamide gels and transferred onto polyvinylidene difluoride (PVDF) membranes. Membranes were blocked with 5% skim milk in Tris-buffered saline, 0.1% Tween 20 (TBST) for 1 h at room temperature (RT) and then incubated overnight at 4°C with an affinity-purified monoclonal mouse antibody against LOTUS (ITM). After the membranes were washed with TBST, they were incubated with horseradish peroxidase-conjugated secondary antibody for 1 h at RT. The bands were visualized using an enhanced chemiluminescence reagent (GE Healthcare) and an ImageQuant LAS 4000 instrument (GE Healthcare). The quantification of each band was performed using the National Institutes of Health image analyzer.

### Behavioral analyses

The hindlimb motor function of each mouse was evaluated weekly using the Basso mouse scale (BMS) up to 42 d after injury (Basso et al., 2006). Two persons blinded to the mouse group performed the behavioral analyses. At 42 d after injury, motor function was also assessed on a rotating rod apparatus (KDS310; Muromachi-Kikai Co., Ltd.) by measuring the amount of time that mice could remain on the rod while it rotated at 10 rotations per minute (rpm). A treadmill gait analysis was performed using the DigiGait System (Mouse Specifics). The stride lengths and stance angles of the hindlimbs were measured on a treadmill at a speed of 6 cm/s, and phase dispersion was analyzed in the DigiGait analyses to provide an indication of limb coordination.

### Electrophysiology

Electrophysiological experiments were performed using a Neuropack S1 MEB_9402_ signal processor (Nihon Kohden; http://www.nihonkohden.co.jp) at 56 d postinjury as previously described (Nori et al., 2011). Mice were anesthetized by intraperitoneal injections of ketamine (60 mg/kg) and xylazine (10 mg/kg), and a C1 laminectomy was performed (*n* = 10, each group). The occipitocervical area of the spinal cord was stimulated, and the signal in the hindlimb was detected by needle electrodes. The active electrode was placed in the quadriceps muscle belly, the reference electrode was placed near the distal quadriceps tendon of the muscle, and the ground electrode was placed in the tail. Stimulation with an intensity of 0.8 mA, duration of 0.2 ms, and interstimulus interval of 1 Hz was used. The latency was measured as the length of time from the stimulation to the onset of the first response wave. The amplitude was measured from the initiation point of the first response wave to its highest point.

### Immunohistochemistry (IHC)

Anesthetized mice were transcardially perfused with saline solution, followed by 4% paraformaldehyde PBS 56 d after injury. Spinal cords were dissected and postfixed in 4% paraformaldehyde for 2 h at RT. Fixed spinal cords were soaked in 10% sucrose in 0.1 M PBS overnight at 4°C, followed by 30% sucrose, embedding in Optimal Cutting Temperature compound (Sakura Finetechnical Co., Ltd.), and freezing as previously described (Nishimura et al., 2013). Samples were sectioned in the sagittal plane at a thickness of 14 μm or the axial plane at a thickness of 20 μm on a cryostat (Leica CM3050 S, Leica Microsystems). Histologic analyses of the sections were performed by hematoxylin-eosin (HE) staining, Luxol fast blue (LFB) staining and IHC. Tissue sections were stained with the following primary antibodies for IHC: anti-human influenza hemagglutinin [HA; rabbit immunoglobulin G (IgG), 1:500; CosmoBio], anti-glial fibrillary acidic protein (GFAP; rabbit IgG, 1:500; Dako, Z0334), anti-neurofilament 200 kDa (NF-H; mouse IgG, 1:500; Millipore, MAB5262), anti-5-HT (goat IgG, 1:500; Immunostar, Inc., 20079), anti-phosphorylated growth-associated protein 43 (p-GAP43; mouse IgG, 1:1000; Wako, 18-10H-9H), anti-neuronal nuclear protein (NeuN; mouse IgG, 1:500; Millipore Bioscience Research Reagents, MAB377), and anti-cleaved caspase-3 (rabbit IgG, 1:500; Cell Signaling, 9661). Then, the sections were incubated with Alexa Fluor-conjugated secondary antibodies (1:1000). For IHC with anti-p-GAP43, a biotinylated secondary antibody (Jackson ImmunoResearch Laboratories Inc.) was used after the tissue was exposed to 1.0% H_2_O_2_ for 30 min at RT to inactivate endogenous peroxidases. Signals were enhanced with a Vectastain ABC kit (Vector Laboratories, Inc.). Nuclei were stained with Hoechst 33258 (10 μg/ml, Sigma-Aldrich). All images were obtained using a fluorescence microscope (BZ-X710; Keyence Co.) and a confocal laser scanning microscope (LSM 700, Carl Zeiss).

### Terminal deoxynucleotidyl transferase-mediated dUTP nick end labeling (TUNEL) staining

Apoptotic cells were detected using the TUNEL method. TUNEL staining was performed using an *In Situ* Cell Death Detection kit, Fluorescein (Roche). At 7 d postinjury, axial sections were costained with TUNEL and Hoechst 33258, and only TUNEL-positive cells that correlated with Hoechst-positive nuclei were counted. The percentage of apoptotic cells was quantified by counting the numbers of TUNEL-positive cells at the margin of the lesion in epicenter axial sections under a 400× objective (*n* = 5, each group).

### Anterograde labeling of the CST

At 42 d postinjury, mice in both groups were injected with biotinylated dextran amine (BDA; MW 10,000; 10% in DW, Invitrogen) to trace the descending CST fibers (*n* = 5, each group). BDA was injected into the right primary motor cortex at the following four sites: 0.5 and 1.5 mm posterior from bregma and 0.5 and 1.5 mm lateral from bregma at a depth of 0.7 mm, with 0.3 μl injected at each site. Two weeks after injection, the mice were sacrificed, and spinal cord samples were sectioned in the axial plane at a thickness of 20 μm on a cryostat. The BDA tracer was visualized by Alexa Fluor 488 conjugated to streptavidin, and histologic analyses were performed.

### Retrograde labeling of the reticulospinal tract

At 42 d postinjury, mice in both groups were injected with Fluoro-Gold (FG; 4%; Fluorochrome, Inc) to retrogradely trace reticulospinal tract fibers (*n* = 5, each group). FG was injected into the lumbar enlargement of the spinal cord at two sites, 0.5 mm on either side of the center with 0.5 μl injected at each site. One week later, the mice were sacrificed, and histologic analyses were performed.

### Quantitative analysis

The spinal cord area was quantified using HE staining of axial sections at the lesion epicenter and 0.5 and 1.0 mm rostral and caudal to the epicenter under a 10× objective (*n* = 5, each group). For the evaluation of the myelinated areas, the LFB-positive areas of axial sections were measured (*n* = 5, each group). To evaluate the lesion area, we measured the GFAP-negative area at the lesion epicenter in midsagittal sections under a 10× objective (*n* = 5, each group). The NF-H- and 5-HT-positive areas in whole axial sections of the lesion epicenter and 2 and 4 mm rostral and caudal to the epicenter were quantified under a 10× objective (*n* = 5, each group). Quantitative analysis of the p-GAP43-positive area was performed using the immunoreactive area of the spinal white matter in axial sections 4 mm caudal to the epicenter under a 20× objective (*n* = 5, each group). CST fibers were evaluated by measuring the BDA-positive area of the dorsal column and ipsilateral and contralateral gray matter in the axial sections every 0.5 mm from 5.0 mm rostral to 2.0 mm caudal to the epicenter under a 20× objective (*n* = 5, each group). For the obtained images, the threshold value for signal detection was set, and the total number of pixels above a certain intensity was measured as the BDA-positive area (Steward et al., 2008). The axon index of the CST was calculated as a ratio to the dCST area 5.0 mm rostral to the epicenter. FG-labeled reticulospinal tract neurons within the reticular formation of the brainstem were quantified in each animal from every three slices of axial sections under a 20× objective (*n* = 5, each group). For analysis of apoptotic cells, cleaved caspase-3-positive nuclei within the automatically captured images of the marginal area of the lesion were counted in each animal from every five images in the axial sections of the epicenter under a 40× objective. These counts were expressed as a percentage of the total nuclei (*n* = 3, each group). All images were quantified using ImageJ (https://imagej.nih.gov/ij/).

### Experimental design and statistical analysis

All data are presented as the mean ± SEM. Unpaired two-tailed Student’s *t* test was used for single comparisons between the control and LOTUS groups. Repeated-measures two-way ANOVA followed by the Tukey–Kramer test was used for BMS scores. Two-way ANOVA followed by the Bonferroni multiple comparisons test was used for the remainder of the analyses. Differences were considered significant at *p* < 0.05 or *p* < 0.01.

## Results

### Characterization of LOTUS overexpression in LOTUS-transgenic mice

**Figure 1.**
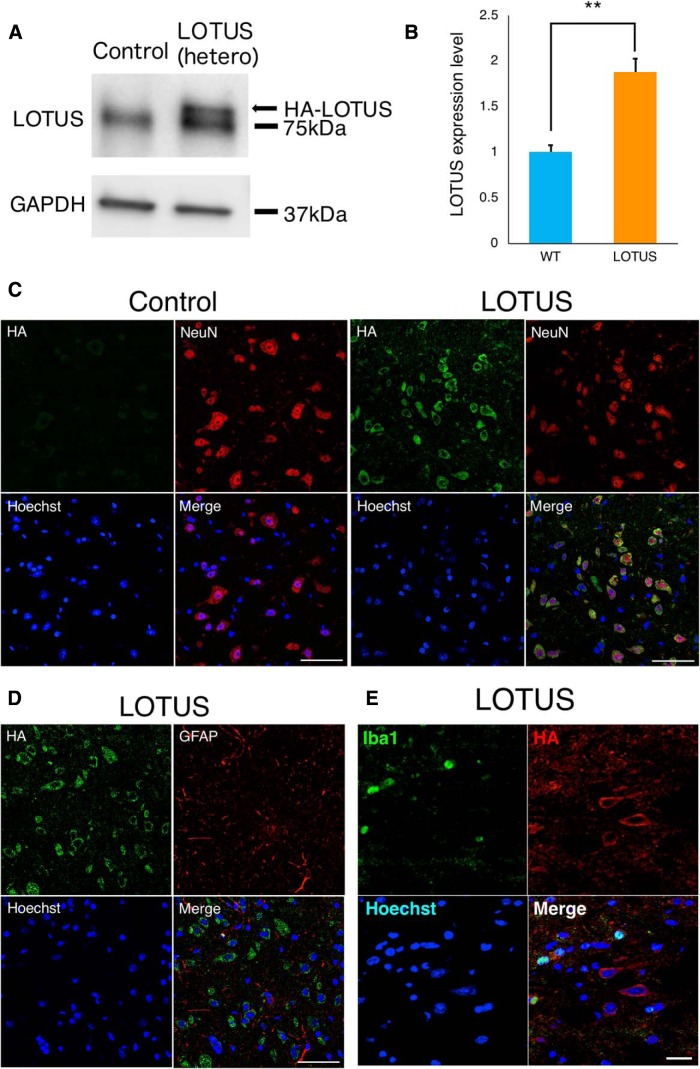
Characterization of LOTUS expression profile in LOTUS-Tg mice. ***A***, Western blot analyses of LOTUS protein expression in the thoracic spinal cord. ***B***, Quantitative analyses of the expression level of LOTUS relative to that of GAPDH. ***C***, Representative images of overexpressed LOTUS labeled with HA in axial sections at the thoracic level in control and LOTUS-Tg mice. Representative images of HA/NeuN/nuclear triple immunostaining. ***D***, Representative images of HA/GFAP/nuclear triple immunostaining in LOTUS-Tg mice. ***E***, Representative images of Iba1/HA/nucleus triple immunostaining in the axial section of the LOTUS group at 7 d postinjury. Values are the mean ± SD; *p < 0.05. Statistical analysis was performed using unpaired two-tailed Student’s *t* test (*n* = 5 each). Scale bars: 50 μm (***C***, ***D***) and 20 μm (***E***).

We used transgenic mice (LOTUS-Tg mice) with heterozygous LOTUS overexpression under the mouse synapsin-1 promoter with a HA tag (Hirokawa et al., 2017). Western blotting of the intact thoracic spinal cord showed a band of overexpressed LOTUS above the 75-kDa band for endogenous LOTUS in LOTUS-Tg mice (Fig. 1*A*). Quantitative analyses revealed that the expression level of LOTUS in LOTUS-Tg mice was nearly twice that in wild-type mice (Fig. 1*B*; *p* = 0.0002). Immunostaining revealed HA-positive cells in the spinal cord of LOTUS-Tg mice but not in that of wild-type mice (Fig. 1*C*). In LOTUS-Tg mice, HA-positive cells were costained with NeuN, a neuronal marker, but not GFAP, an astrocyte marker (Fig. 1*C*,*D*), indicating that overexpressed LOTUS was expressed in neuronal cells. Furthermore, histologic analysis demonstrated that the overexpressed HA-tagged LOTUS was not expressed in ionized calcium-binding adapter molecule 1 (Iba1)-positive microglia cells (Fig. 1*E*). However, there was no significant difference in the appearance (body weight) or locomotor behaviors assessed by the BMS score, rota-rod test and DigiGait analysis. The baseline BMS score of intact control and LOTUS-Tg mice was nine, and the cutoff value in the rota-rod test was 120 s (*n* = 5, each group). The stride lengths, stance angles of the hindlimbs and phase dispersions in DigiGait analyses were as follows: preinjury: control versus LOTUS; stride length: 4.36 ± 0.57 versus 4.49 ± 0.62; stance angle: 14.03 ± 4.69 versus 14.72 ± 8.44; phase dispersions: FL-HR; 0.0096 ± 0.018 versus 0.0034 ± 0.038; FR-HL: 0.011 ± 0.034 versus 0.0019 ± 0.026; FL-FR: 0.48 ± 0.028 versus 0.48 ± 0.042; HL-HR: 0.48 ± 0.05 versus 0.49 ± 0.063; FR-HR: 0.48 ± 0.041 versus 0.5 ± 0.039; FL-HL: 0.48 ± 0.034 versus 0.49 ± 0.066; no significant difference, *n* = 4, each group. Similarly, neuronal fibers labeled by 5-HT and NF-H between LOTUS-Tg and wild-type mice showed no significant differences (data not shown).

### LOTUS suppresses spinal cord atrophy and protects myelinated neuronal fibers against injury

**Figure 2.**
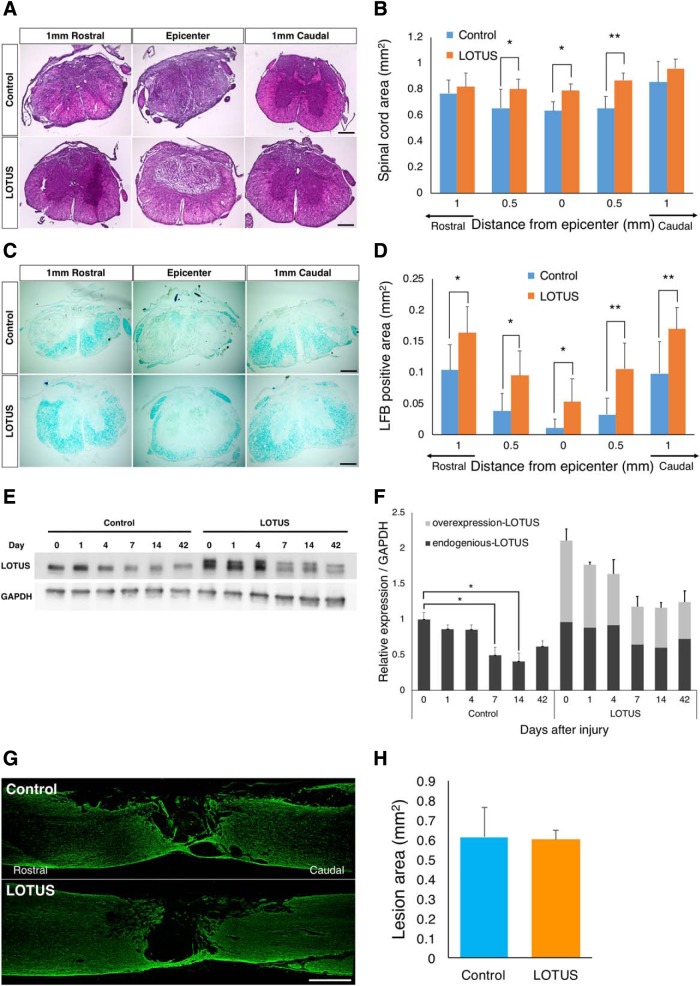
LOTUS minimizes spinal cord atrophy and preserves myelinated nerve fibers after SCI. ***A***, Representative images of HE staining in axial sections from 1 mm rostral to 1 mm caudal to the epicenter 56 d after SCI. ***B***, Quantitative analysis of the spinal cord area. ***C***, Representative images of HE staining in axial sections from 1 mm rostral to 1 mm caudal to the epicenter 56 d after SCI. ***D***, Quantitative analysis of the LFB-positive myelinated area. ***E***, Western blot analyses of endogenous and overexpressed LOTUS protein expression in the injured spinal cord. ***F***, Quantitative analyses of the time course changes of the expression level of endogenous and overexpressed LOTUS relative to that of GAPDH. ***G***, Representative images of immunostaining for GFAP in sagittal sections 56 d after SCI. ***H***, Quantitative analysis of the lesion area size surrounded by reactive astrocytes. Values are the mean ± SD; *p < 0.05, **p < 0.01. Statistical analysis was performed using two-way ANOVA, followed by the Bonferroni multiple comparisons test in ***B***, ***D***, ***F*** and unpaired two-tailed Student’s *t* test in ***H*** (*n* = 5 each). Scale bars: 200 μm (***A***, ***C***) and 500 μm (***G***).

To evaluate the effects of pan-neuronally overexpressed LOTUS on the injured spinal cord, we analyzed axial sections using HE staining at 56 d postinjury. Quantitative analyses revealed a significantly larger spared volume of the spinal cord area at the injury epicenter and 0.5 mm caudal to the epicenter in the LOTUS group than in the control group (Fig. 2*A*,*B*; df = 8, 0.5 mm rostral *p* = 0.024; epicenter *p* = 0.019; 0.5 mm caudal *p* = 0.001). To assess the effect of LOTUS on the preservation of myelin, we examined LFB-stained axial sections. The LFB-positive myelinated area in the injured spinal cord was significantly more preserved from 1 mm rostral to caudal to the epicenter in the LOTUS group than in the control group (Fig. 2*C*,*D*; 1 mm rostral *p* = 0.01; 0.5 mm rostral *p* = 0.014; epicenter *p* = 0.04; 0.5 mm caudal *p* = 0.002; 1 mm caudal *p* = 0.002). To clarify the effects of overexpressed LOTUS on the suppression of atrophy and preservation of myelinated neuronal fibers, we determined the time course changes in the expression level of endogenous and overexpressed LOTUS in the injured spinal cord using Western blotting. Endogenous LOTUS expression gradually decreased in the acute phase after SCI, and a significant decrease was observed at 7 and 14 d postinjury. For the overexpressed LOTUS, the expression level was not elevated after injury, which suggested that the existing expression level of total LOTUS (endogenous and overexpressed) was sufficient for the neuroprotective effect in LOTUS-Tg mice (Fig. 2*E*,*F*; day 0 vs day 7 in the control group *p* = 0.04; day 0 vs day 7 in the control group *p* = 0.023; the control group vs the LOTUS group at every time point *p* < 0.01). Immunostaining for the astrocytic marker GFAP was performed to measure the size of the lesion area surrounded by reactive astrocytes 56 d postinjury. However, there were no significant differences between the two groups, implying that LOTUS overexpression did not affect glial scar size (Fig. 2*G*,*H*).

### LOTUS reduced axonal dieback of CST fibers but did not promote long-distance elongation across the lesion following SCI

**Figure 3.**
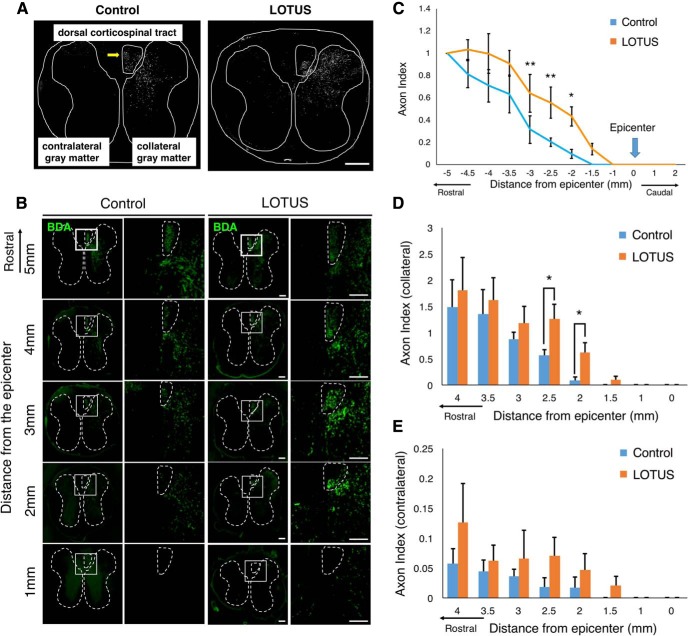
LOTUS reduced axonal dieback in CST fibers following SCI. ***A***, A schematic illustration of the dCST and collateral/contralateral CST in axial sections at 2.5 mm rostral to the epicenter 56 d after SCI. ***B***, Representative images of CST fibers labeled with BDA in axial sections from 5 mm to 1 mm rostral to the epicenter at 56 d after SCI. The dCST and collateral/contralateral gray matter areas are surrounded by a dotted line. Enlarged images of the area in the white box are shown, and dCST areas were similarly surrounded. ***C***, Quantitative analysis of the dCST fibers (relative to the fibers at 5 mm rostral) from 5 mm rostral to the epicenter to sites caudal to the epicenter. ***D***, ***E***, Quantitative analyses of the axon index in the ipsilateral/contralateral gray matter CST relative to that in dCST 5 mm rostral to the epicenter. Values are the mean ± SD; *p < 0.05. Statistical analysis was performed using two-way ANOVA, followed by the Bonferroni multiple comparisons test (*n* = 5 each) in ***C–E***. Scale bars: 200 μm (***A***) and 100 μm (***B***).

Although the CST is the most important descending pathway for motor function in humans (Lemon, 2008), the effects of NgR1 on CST fibers following SCI are controversial. To examine the influence of LOTUS pan-neuronal overexpression on CST fibers, we injected an anterograde tracer, BDA, into the primary motor cortex of mice in both groups and sacrificed the mice for histologic analysis two weeks after injection. BDA labeling confirmed that CST fibers were present in the dorsal column (dCST) and ipsilateral/contralateral gray matter (ipsilateral/contralateral CST; Fig. 3*A*; Kim et al., 2003). The dCST extended significantly further into the rostral sites (from 3.0 to 1.0 mm rostral to the epicenter) in the LOTUS group than in the control group (Fig. 3*B*,*C*; 3.0 mm rostral *p* = 0.006; 2.5 mm rostral *p* = 0.008; 2.0 mm rostral *p* = 0.033). The dCST in the control group was observed up to 2 mm rostral from the injury epicenter, whereas the BDA-positive area in the LOTUS group was observed up to 0.5 mm rostral, closer to the epicenter, than that in the control group. The dCST was not detected caudal to the epicenter in either of the groups (Fig. 3*B*,*C*). An increase in ipsilateral and contralateral CST fibers was observed with LOTUS overexpression in the rostral sites, with a significant difference in fibers seen in the ipsilateral CST 2 and 2.5 mm rostral to the epicenter (Fig. 3*D*,*E*; 2.5 mm rostral *p* = 0.026; 2 mm rostral *p* = 0.048). These results suggested that LOTUS promoted the increase in the CST fibers rostrally anterior to the lesion due to reduced axonal dieback anterior to the lesion. However, LOTUS could not promote long-distance extension across the lesion area.

### LOTUS promotes an increase in reticulospinal neurons that project axons caudal to the injury site

**Figure 4.**
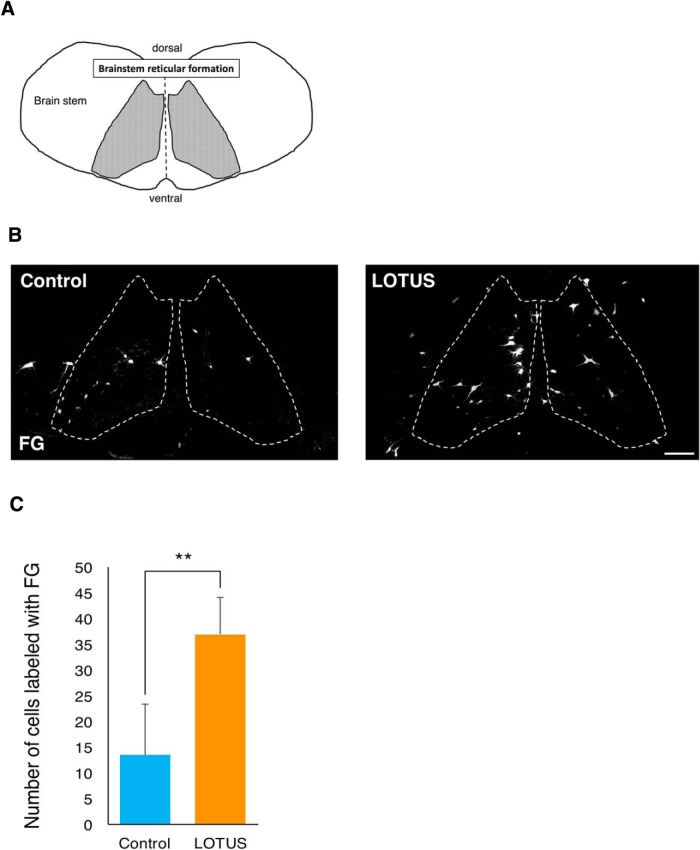
LOTUS increases reticulospinal neurons projecting axons caudal to the lesion following SCI. ***A***, A schematic illustration of the brainstem reticular formation. ***B***, Representative images of reticular nucleus neurons retrogradely labeled with FG. ***C***, Quantitative analysis of the number of cells labeled with FG. Values are the mean ± SD; *p < 0.05, **p < 0.01. Statistical analysis was performed using unpaired two-tailed Student’s *t* test (*n* = 5 each in ***C***). Scale bars: 100 μm (***B***).

Although CST fibers failed to regenerate beyond the lesion, we also traced other descending pathways. According to previous studies, the reticulospinal tract may be more important than the CST for motor function in rodents (Ballermann and Fouad, 2006; Lemon, 2008). To trace reticulospinal tract fibers, we injected the retrograde tracer, FG, into the spinal cord caudal to the injury site in both groups of mice. Then, we sacrificed the mice one week after injection and histologically analyzed the reticular formations in the brainstem (Fig. 4*A*). This analysis revealed a significant increase in the number of FG-labeled reticular nucleus neurons in the LOTUS group (Fig. 4*B*,*C*; LOTUS group; 36.93 ± 7.06 vs control group; 13.41 ± 9.88, *p* = 0.004). Together, these data demonstrated that a greater number of reticulospinal neurons project axons caudal to the lesion site following SCI in the LOTUS group than in the control group.

### LOTUS contributes to an increase in neuronal fibers in the injured spinal cord

Following the tracer experiments, we evaluated the effect of pan-neuronal LOTUS overexpression on neuronal fibers in the injured spinal cord through immunohistological analyses. Fifty-six days postinjury, immunostaining was performed using antibodies against neurofilament heavy chain (NF-H) and 5-HT, markers for neuronal fibers and the serotonergic raphespinal tract, respectively. The serotonergic raphespinal tract, one of the major descending pathways, is especially important for the motor functional circuitry of hindlimbs (Bregman, 1987; Saruhashi et al., 1996; Kim et al., 1999).

**Figure 5.**
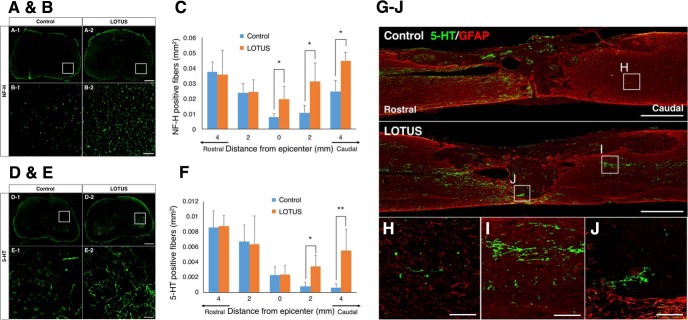
LOTUS promotes the regeneration of NF-H- and 5-HT-positive fibers. ***A***, Representative images of immunostaining for NF-H in axial sections at 4 mm caudal to the epicenter 56 d after SCI. ***B***, Enlarged images of NF-H-positive fibers of the area in the white box in ***A***. ***C***, Quantitative analysis of NF-H-positive fibers from 4 mm rostral to 4 mm caudal to the epicenter. ***D***, Representative images of immunostaining for 5-HT in axial sections at 4 mm caudal to the epicenter 56 d after SCI. ***E***, Enlarged images of 5-HT-positive fibers of the area in the white box in ***D***. ***F***, Quantitative analysis of the 5-HT-positive fibers from 4 mm rostral to 4 mm caudal to the epicenter. ***G***, Representative images of immunostaining for 5-HT/GFAP in sagittal sections 56 d after SCI. ***H–J***, Enlarged images of the area in the white box in ***G***. Values are the mean ± SD; *p < 0.05. Statistical analysis was performed using two-way ANOVA, followed by the Bonferroni multiple comparisons test (*n* = 5 each). Scale bars: 200 μm (***A***, ***D***), 50 μm (***B***, ***E***, ***H–J***), and 500 μm (***G***).

The NF-H-positive area in the axial section of the LOTUS group was significantly larger at and beyond the epicenter than that of the control group (Fig. 5*B*,*C*; epicenter *p* = 0.035; 2 mm caudal *p* = 0.037; 4 mm caudal *p* = 0.04). The 5-HT-positive area was also significantly greater in the LOTUS group from 2 mm to 4 mm caudal to the epicenter (Fig. 5*D–F*; 2 mm caudal *p* = 0.04; 4 mm caudal, *p* = 0.001). In the midsagittal sections of the control group, almost no 5-HT-positive fibers extending to the caudal site beyond the lesion were observed. By contrast, in the LOTUS group, many fibers were seen at the caudal site, and fibers were also detectable in the lesion epicenter (Fig. 5*G–J*).

### LOTUS enhances raphespinal tract regeneration

Although we revealed that LOTUS overexpression led to an increase in NF-H-positive and raphespinal tract fibers 56 d postinjury, whether this increase resulted from neuronal protection against secondary injury or the regeneration of neuronal fibers at the chronic stage was unclear. To address this issue, we examined 5-HT-positive raphespinal tract fibers in more detail.

**Figure 6.**
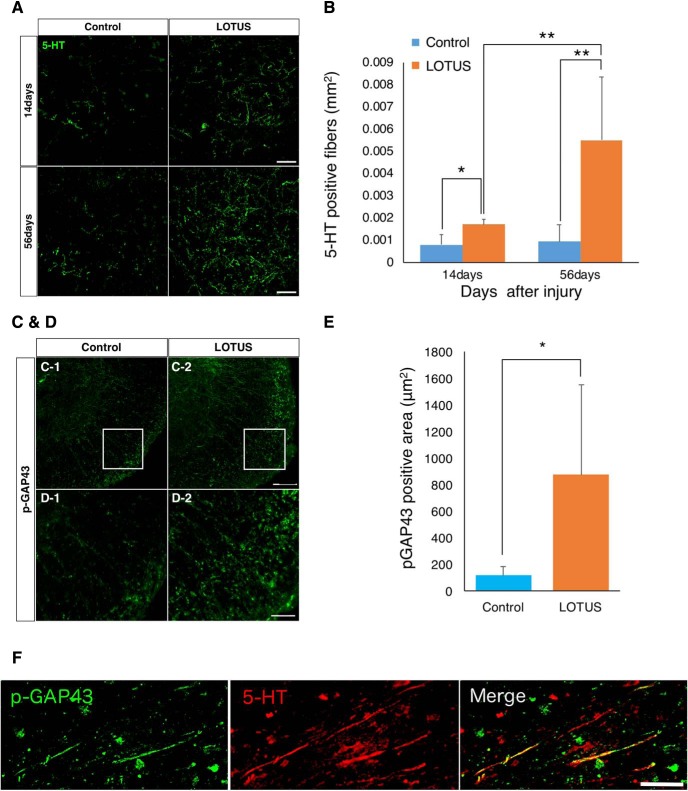
LOTUS promotes the regeneration and protection of 5-HT-positive fibers caudal to the epicenter after SCI. ***A***, Representative images of immunostaining for 5-HT in axial sections at 4 mm caudal to the epicenter 14 and 56 d after SCI. ***B***, Quantitative analysis of 5-HT-positive fibers 14 and 56 d after SCI. ***C***, Representative images of immunostaining for p-GAP43 in axial sections at 4 mm caudal to the epicenter 56 d after SCI. ***D***, Enlarged images of NF-H-positive fibers in the area surrounded by the white box in ***C***. ***E***, Quantitative analysis of the p-GAP43-positive fibers. ***F***, Representative images of double immunostaining for p-GAP43 and 5-HT. Values are the mean ± SD; *p < 0.05. Statistical analysis was performed using two-way ANOVA, followed by the Bonferroni multiple comparisons test in ***B*** and unpaired two-tailed Student’s *t* test in ***E*** (*n* = 4 each at day 14, *n* = 5 each at day 56 after SCI in B, *n* = 4 each in ***E***). Scale bars: 50 μm (***A***, ***D***, ***F***) and 100 μm (***C***).

We immunohistochemically analyzed raphespinal tract fibers in axial sections 4 mm caudal to the epicenter at 14 and 56 d postinjury (Fig. 6*A*). At 14 d postinjury, a larger 5-HT-positive area was observed in the LOTUS group than in the control group (*p* = 0.035). Notably, a significant increase was detected between 14 and 56 d postinjury in the LOTUS group (14 vs 56 d *p* = 0.002; control vs LOTUS at 56 d *p* = 0.000), while this trend was not observed in the control group (*p* = 0.827; Fig. 6*B*). Taken together, these results indicated that LOTUS enhanced the regeneration of raphespinal tract fibers.

Furthermore, we performed immunostaining of phosphorylated growth-associated protein 43 (pGAP43), a growth cone marker that provides an indication of nerve regeneration (Fukura et al., 2000; Fig. 6*C*,*D*). In axial sections 4 mm caudal to the epicenter at 56 d postinjury, the pGAP43-positive area was significantly larger in the LOTUS group than in the control group (Fig. 6*E*; *p* = 0.023). Double immunostaining with 5-HT and pGAP43 antibodies in the midsagittal sections of the LOTUS group showed partial coexpression of pGAP43 in 5-HT-positive fibers (Fig. 6*F*). These results strongly suggested that LOTUS promoted the regeneration of the raphespinal tract following injury.

### LOTUS suppresses injury-induced apoptotic cell death

**Figure 7.**
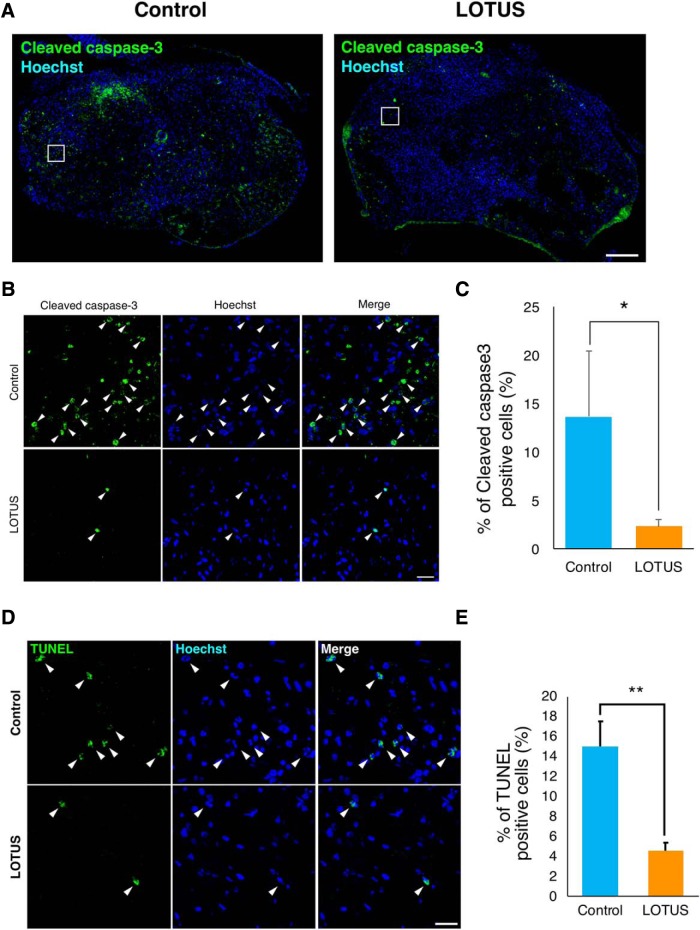
LOTUS suppresses apoptotic cell death at the acute injury phase. ***A***, Representative images of apoptotic cells immunostained for cleaved caspase-3 in axial sections at the epicenter 7 d after SCI. ***B***, Enlarged images of the margin of the epicenter (white box in ***A***; green: cleaved caspase-3, blue: nucleus). The arrows indicate apoptotic nuclei stained for cleaved caspase-3. ***C***, Quantitative analysis of the percentage of apoptotic cells. ***D***, Representative images of TUNEL staining in axial sections at the epicenter 7 d after SCI. ***E***, Quantitative analysis of the percentage of TUNEL-positive cells. Values are the mean ± SD; *p < 0.05, **p < 0.01. Statistical analysis was performed using unpaired two-tailed Student’s *t* test (*n* = 3 each). Scale bars: 200 μm (***A***), 50 μm (***B***), and 20 μm (***D***).

Our data have demonstrated that LOTUS overexpression minimizes spinal cord atrophy, preserves the myelinated area, and promotes the regeneration of raphespinal tract fibers following early injury (Figs. 2*A*,*C*, 6*A*,*B*), suggesting that LOTUS protects spinal cord tissue, including neuronal fibers, following injury. Immunostaining of cleaved caspase-3, a marker for apoptosis, revealed apoptotic cells at the margin of the lesion 7 d postinjury in both groups (Fig. 7*A*), and quantitative analyses revealed that the LOTUS group expressed significantly fewer apoptotic cells (Fig. 7*B*,*C*; *p* = 0.016). Additionally, we performed TUNEL staining at 7 d postinjury, which showed a decrease in TUNEL-positive apoptotic cells in the LOTUS group (Fig. 7*D*,*E*; *p* = 0.004). These results suggested that apoptosis at the acute phase of SCI was prevented by LOTUS overexpression.

**Figure 8.**
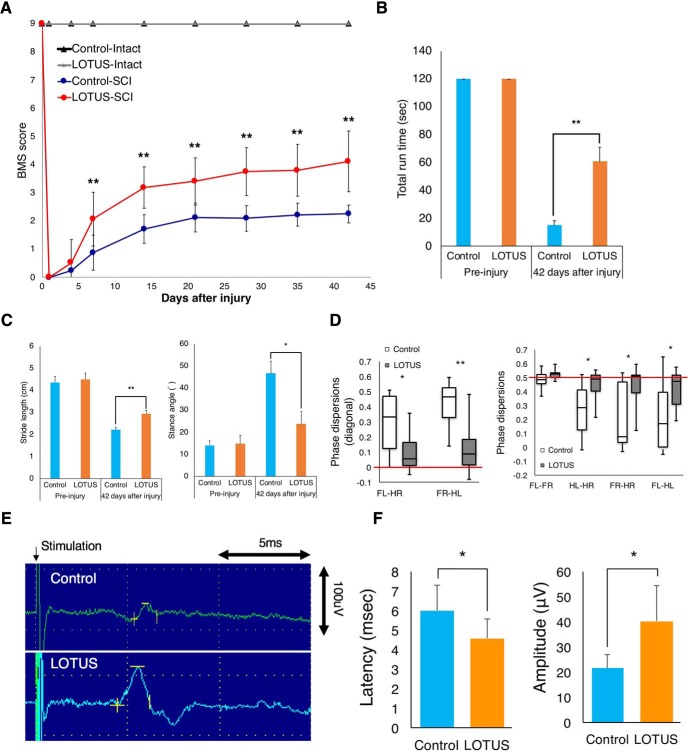
Evaluation of motor functional recovery and MEP waves. ***A***, Hindlimb motor function was evaluated weekly for six weeks after SCI by BMS scores in the control and LOTUS groups (intact model, control group; *n* = 5, LOTUS group; *n* = 5, SCI model, control group; *n* = 16, LOTUS group; *n* = 20). ***B***, The rota-rod test was performed 42 d after SCI, and total run time was quantitatively analyzed (preinjury; *n* = 5 each, control group; *n* = 9, LOTUS group; *n* = 10). ***C***, Treadmill gait analyses using a DigiGait System were examined 42 d after SCI, and quantitative analyses of the stride length and stance angle were performed (preinjury; *n* = 5 each, control group; *n* = 9, LOTUS group; *n* = 14). ***D***, Hindlimb coordination was analyzed by phase dispersions in DigiGait analyses (control group; *n* = 9, LOTUS group; *n* = 14). ***E***, Electrophysiological analysis of MEP waves was performed 56 d after SCI, and representative images are shown (*n* = 10 each). ***F***, Quantitative analyses of the latency and amplitude were performed (control group; *n* = 5, LOTUS group; *n* = 8). Values are the mean ± SD; *p < 0.05, **p < 0.01. Statistical analysis was performed using unpaired two-tailed Student’s *t* test in the rota-rod test, DigiGait analyses, MEP analysis and two-way repeated-measures ANOVA with Tukey’s test in the analysis of the BMS score.

### LOTUS promotes the recovery of motor function and nerve conduction

Motor functional recovery following SCI was assessed using the BMS scoring system, rota-rod test and DigiGait footprint analysis. The BMS scores in the LOTUS group were significantly better than those in the control group at one week following SCI and thereafter (Fig. 8*A*). Furthermore, the LOTUS group exhibited a significantly longer total run time in the rota-rod test 42 d post-SCI (Fig. 8*B*; *p* = 0.001). In the treadmill gait analyses using DigiGait, a significantly longer hindlimb stride length and narrower hindlimb stance angle were seen in the LOTUS group than in the control group 42 d postinjury (Fig. 8*C*; stride length *p* = 0.004; stance angle *p* = 0.013). Phase dispersion, an indicator of coordination measured using DigiGait analysis, showed that the LOTUS group exhibited significantly better hindlimb coordination than did the control group (Fig. 8*D*). Although there was no significant difference, electrophysiological analysis revealed motor evoked potential (MEP) waves in 80% of the LOTUS group and 50% of the control group at final follow-up. Moreover, we found a significantly shorter MEP latency and larger MEP amplitude in the LOTUS group (Fig. 6*E*,*F*; latency *p* = 0.049; amplitude *p* = 0.017), indicating a potential benefit of LOTUS pan-neuronal overexpression for the improvement in nerve conduction.

## Discussion

In this study, we evaluated the therapeutic benefits of LOTUS pan-neuronal overexpression in the injured spinal cord using a clinically relevant contusive SCI model. Here, we showed that LOTUS has a neuroprotective effect on the spinal cord through reducing cellular apoptosis during the acute phase following SCI. Histologic evaluation of descending motor fiber tracts revealed that LOTUS overexpression enhanced raphespinal axonal regeneration across the lesion area to the caudal site during the chronic stage, increased reticulospinal neurons projecting axons caudal to the lesion and reduced axonal dieback of the CST rostrally anterior to the lesion. However, the long-distance elongation of the CST was not observed beyond the edge of the caudal lesion site. The neural preservation and regeneration activity of LOTUS may have contributed to the significant motor functional recovery observed in these mice. Thus, administration of LOTUS in the treatment of SCI could be a promising strategy through promoting endogenous restoration and locomotor improvement.

Detailed histologic analyses revealed remarkable effects of LOTUS overexpression on raphespinal tract regeneration. Although the raphespinal tract is known for its modulatory effects on sensory activity (Mason, 2001), it can function as a detour circuit, connecting to the motor circuit after SCI (Liang et al., 2015). In fact, a previous study using the serotonergic neurotoxin 5,7-dihydroxytryptamine showed that raphespinal regeneration following SCI significantly contributed to functional recovery in NgR1-lacking mice (Kim et al., 2004) and Semaphorin 3A (Sema3A) inhibitor-treated rats (Kaneko et al., 2006), thereby substantiating our results that demonstrated a favorable effect of the NgR1 antagonist on the tract. Furthermore, Hirokawa et al. (2017) reported that pan-neuronally LOTUS-overexpressing transgenic mice obtained significantly enhanced motor functional recovery with the increase in 5-HT positive raphespinal tract fibers after injury, whereas LOTUS-deficient mice obtained less functional recovery than did control mice with the decrease in raphespinal tract fibers. These results suggested that raphespinal tract regeneration was relevant to locomotor recovery, and the present study also supported this hypothesis.

Alternatively, a retrograde tracing study demonstrated that LOTUS expression may contribute to the increase in reticulospinal neurons that project axons caudal to the lesion postinjury. No study has clarified the relationship between the regeneration of the reticulospinal tract and NgR1 in the injured spinal cord. This tract is implicated in central pattern generators responsible for locomotion generation (Fink and Cafferty, 2016) and mediates the fastest descending excitation observed in motoneurons (Alstermark et al., 2004). It is possible that LOTUS increased reticulospinal tract fibers, which may be intimately associated with neurologic restoration after SCI. Further studies of the phenotype and time course changes postinjury are needed to elucidate the regenerative effect of LOTUS on the reticulospinal tract.

In pan-neuronally LOTUS-overexpressing mice, we observed a reduction in cellular apoptosis and an increase in neuronal fiber preservation in the subacute phase of SCI. This neural preservation led to an earlier motor functional recovery in the BMS score in LOTUS-overexpressing mice than in control mice. Similarly, administration of Nogo-66 antagonist peptide (NEP1-40), an NgR1 antagonist, also showed neuroprotective effects via inhibition of neuronal apoptosis after cerebral ischemic injury (Wang et al., 2008). However, little is known about the mechanism by which NgR1 signaling affects cellular apoptosis after CNS injury. To clarify this question, a previous study revealed that activated NgR1 following brain ischemia led to activation of the Rho-A and ROCK pathways, which induced neuronal apoptosis (Zhang et al., 2007). Furthermore, administration of a Rho-A inhibitor suppressed apoptosis in a SCI model (Dubreuil et al., 2003). The Rho-A inhibitor blocked the conversion of Rho-guanosine diphosphate (GDP) to Rho-guanosine triphosphate (GTP) and activation of p75 neurotrophin receptor (p75^NTR^), thereby blocking p75^NTR^-dependent apoptosis. Therefore, NgR1 may play an important role in SCI-induced apoptosis.

In the present study, we evaluated the efficacy of LOTUS overexpression in neuronal protection, axonal regeneration and functional recovery using a contusive SCI model. Transection models are most often used to examine axonal extension after SCI because of the resulting clearly defined lesion area, especially in most previous reports that evaluated axonal regeneration through NgR inhibition. However, these models do not represent the common injury mechanisms found in the clinical setting (Forgione et al., 2017). SCI is largely caused by contusion and compression injuries, which are followed by primary and secondary injury phases. Therefore, selecting a more clinically relevant contusion model is extremely important for preclinical trials when evaluating the protective and regenerative effects of injury during the inflammatory phase (Kwon et al., 2011).

In conclusion, we have demonstrated that pan-neuronal LOTUS overexpression enhances neuronal protection, axonal regeneration and functional recovery after a more clinically relevant contusive SCI model. LOTUS exerts a strong inhibitory action against NgR1 and may represent a therapeutic strategy for SCI.

## Synthesis

Reviewing Editor: Jeffery Twiss, University of South Carolina Decisions are customarily a result of the Reviewing Editor and the peer reviewers coming together and discussing their recommendations until a consensus is reached. When revisions are invited, a fact-based synthesis statement explaining their decision and outlining what is needed to prepare a revision will be listed below. The following reviewer(s) agreed to reveal their identity: Jennifer Dulin. Note: If this manuscript was transferred from JNeurosci and a decision was made to accept the manuscript without peer review, a brief statement to this effect will instead be what is listed below.

This revised manuscript forwarded from J Neurosci extends earlier work on LOTUS by using a contusion SCI model and examining additional descending spinal tracts. The authors have addressed many points of the previous J Neurosci reviewers, but there are a number of lingering points. Numerous strategies similar to LOTUS overexpression that target NgR1 or receptor signaling have been developed and tested extensively for years, but different labs observed inconsistent effects on regeneration and functional recovery. Nonetheless, this manuscript could be considered as replication of previous findings with LOTUS in a new model, so in this way it is appropriate to consider for eNeuro. Though the reviewers did not feel that additional experiments were necessarily needed for further consideration, both noted that better evidence for functional recovery would strengthen the manuscript.

As the paper is currently written, several conclusions drawn are erroneous due to over or misinterpretation of results and/or attributing causation to correlation (see specific comments below). Consequently, the manuscript needs considerable revision. Wording for some sentences is also confusing with awkward English usage, which complicates review of this work and needs to be fully addressed in a revision.

Reviewers expressed concerns over statistics. Specifically, the authors used unpaired t-test in most analyses, where one- or two-way ANOVA would be appropriate. This is important because the conclusions rely greatly on the statistical differences.

Specific points:

Lines 126-127, 135-139 - Numerical behavior scores should be moved to the Results section.

Lines 145-147 - “The occipitocervical area of the spinal cord was stimulated through the hindlimb by needle electrodes”. The spinal cord was stimulated through the hindlimb? Please correct.

Line 250 - please change “HA positive LOTUS” to “HA-tagged LOTUS”.

Line 252 - please change “assed” to “assessed”.

Lines 431-448 - This paragraph is unnecessary. It is generally accepted that the CST is more important for fine motor function of forelimbs, compared to hindlimb function. This is not a new idea. Importantly, your study did not directly test the idea that CST function is mediating any of the functional recovery. Suggest that the authors remove this paragraph.

Line 449 - Change “inhibition” to “reduction”.

Lines 463-466 - There is nothing in the study indicating any role for the Rho/ROCK pathway. Neuroprotection can be mediated by many different signaling pathways. Suggest that the authors remove this sentence.

Lines 480-482 - A correlation of increased raphespinal and reticulospinal axon density with improved hindlimb motor function does not imply causation. There are many, many other neural circuits that were not assessed in this study. The authors did not present any direct test for the role of these tracts in the observed recovery. Suggest that they remove this sentence.

Fig 2A-B - The panel of H&E staining in 2A depicts a larger lesion size in the LOTUS group than in the control group. Is this a representative image? What is “Spinal cord area” and what is the biological meaning of this metric?

Figure 3 - Please provide more details about how the measurement of “BDA-positive area” was performed in this experiment. Does this mean the total number of pixels above some intensity threshold? The total number of axons? Or the entire area of the region of the cord containing the CST? The total number of axons labeled with BDA is the most biologically meaningful way to normalize data, and the authors should re-normalize data to this number.

There is always animal-to-animal variability in the number of CST axons that are actually traced following intracortical injection of BDA. In panel 3B, a concern is that more dCST axons in the main tract are labeled in the LOTUS animal than in the WT animal, which could lead to skewing of the data. The authors should include an image of BDA labeling from these same representative animals at 5 mm rostral to the epicenter, the point that all other data is being normalized to. Also, please draw some dotted line or other border around the dCST in 3B for each individual panel so the area containing the dCST (not just the BDA+ CST area but the entire dCST tract is obvious.

The observation that CST fibers are found at closer distances to the epicenter in LOTUS group (0.5mm rostral) versus CST fibers in the WT group (2 mm rostral) cannot be interpreted as regeneration without actual evidence of axon regeneration. It is much more likely that this result is attributable to less axonal dieback and not true regeneration. The only way to distinguish between these possibilities is by performing a time course study similar to what was done in Figure 6. Additional experiments are probably beyond the scope of the current study so it is important to state clearly that this difference could be due to reduced dieback. The authors cannot definitively claim that this is axon regeneration, and you have no evidence of regeneration.

Figure 4 - The authors conclude from the reticulospinal tracing data, “Together, these data demonstrated that LOTUS overexpression increased the number of reticulospinal tract fibers across the injury site after SCI”. This is not necessarily true because the number of fibers was not quantified. For example, consider the possibility that branching of RT fibers is different in the spinal cord of LOTUS animals versus wild type animals. The correct interpretation of these data is that there are a greater number of reticulospinal neurons that project axons caudal to the lesion site in LOTUS animals.

Figure 5 - It is clear in panel 5G (top) that the 5-HT antibody is staining nonspecific debris within the lesion epicenter. For the analysis of 5-HT staining within the lesion epicenter, how was nonspecific staining excluded from the quantification?
